# Hip joint range of motion is restricted by pain rather than mechanical impingement in individuals with femoroacetabular impingement syndrome

**DOI:** 10.1007/s00402-021-04185-4

**Published:** 2021-09-28

**Authors:** Josefine E. Naili, Anders Stålman, Anders Valentin, Mikael Skorpil, Lars Weidenhielm

**Affiliations:** 1 Department of Women’s and Children’s Health Karolinska Institutet, Motoriklab, Q2:07, Karolinska Universitetssjukhuset, 171 76 Stockholm, Sweden; 2 Department of Molecular Medicine and Surgery, Stockholm Sports Trauma Research Center, Karolinska Institutet, 171 76 Stockholm, Sweden; 3 Capio Artro Clinic, Sophiahemmet, 114 28 Stockholm, Sweden; 4 Praktikertjänst Ortopedi, 117 63, Stockholm, Sweden; 5 Department of Molecular Medicine and Surgery, Karolinska Institutet, 171 76 Stockholm, Sweden

**Keywords:** Femoroacetabular impingement, Computed tomography, Range of motion, 3D simulation, 3D motion analysis

## Abstract

**Introduction:**

Discerning whether range of motion (ROM) is restricted by morphology or other pain sources is challenging in patients with femoroacetabular impingement syndrome (FAIS). Computed tomography (CT) motion simulation provides a hypothetical ROM based on morphology. This study aimed to explore associations between ROM measured using CT motion simulation and maximum passive ROM measured clinically using three dimensional (3D) motion analysis in patients with FAIS, prior to and post arthroscopic hip surgery.

**Materials and methods:**

Eight males with FAIS (in total 12 hip joints) were included in this explorative feasibility study. Participants were examined using CT according to a low-dose protocol prior to and 7-months post arthroscopic surgery. Software was used to simulate at which ROM the impingement would occur. With the hip in 90 degrees’ flexion, maximum passive range of internal hip rotation, and maximum passive internal hip rotation coupled with adduction was examined clinically using 3D motion analysis pre- and postoperatively. Spearman rank correlation coefficients and linear regressions examined associations between methods.

**Results:**

Preoperatively, the correlation between maximum internal hip rotation measured using CT motion simulation and 3D motion analysis was strong (*r* = 0.71, *p* = 0.009). Linear regressions demonstrated that maximal internal rotation measured using CT motion simulation was predominantly larger than when measured using 3D motion analysis. Postoperatively, and when maximum internal rotation was coupled with adduction, no correlations were found between the two methods.

**Conclusions:**

The hypothetical morphology restricted ROM is larger than clinically assessed pain restricted ROM, both prior to and post hip arthroscopy. These findings suggest that ROM is restricted by pain rather than mechanical, morphology-based impingement in individuals with FAIS.

## Introduction

Femoroacetabular impingement (FAI), is a condition where the bones of the hip joint do not have a matching shape. Cam impingement or deformity, refers to a cam effect initiated by a non-spherical femoral head rotating inside the acetabulum [1], while pincer impingement involves focal or general over coverage by the acetabulum of the femoral head [2]. During movement this mismatch may lead to joint friction and reduced range of motion (ROM) [1, 3, 4]. Depending on the severity of the morphologic mismatch and an individual’s activity the condition is associated with cartilage damage [5], eventually causing pain and femoroaceatabular impingement syndrome (FAIS) [1, 3]. It is important to remember that morphologic features and labral injuries are common also in asymptomatic individuals [6]. The specificity for radiographic imaging signs in predicting risk for developing FAIS is poor, since cam changes have been documented in 55% of asymptomatic athletes and 23% in an asymptomatic general population [6, 7].

The main symptoms of FAIS is motion-related or position-related pain in the hip or groin [8]. The onset of pain is usually gradual, without previous known trauma. In addition, pain may also be manifested in the back, buttock or thigh, and patients may also describe restricted ROM, stiffness, clicking, catching or locking sensations [8]. Since these symptoms are similar to those of several differential diagnoses, receiving the diagnosis ‘Femoroacetabular Impingement Syndrome’ often takes time, which potentially delays appropriate treatment. Today, FAIS is diagnosed based on medical history and findings from clinical examination and radiography [8]. Typically, the medical history reveals restricted ROM in deep end range hip flexion, combined with internal rotation and hip adduction [3, 8]. In clinical practice, ROM is routinely measured using goniometers, limiting measures to only include one plane at a time. Three-dimensional (3D) motion analysis is a method based on biomechanical models that use external skin markers to define a local coordinate system to each skeletal segment which enables calculations of joint kinematics in all three planes [9].

Computed tomography (CT) provides additional information, compared to radiography, including 3D information of the joint. In 2007, a clinical pilot study examined a CT based method (‘HipMotion’) for noninvasive 3D assessment of FAI [10]. HipMotion was found to be a reliable, accurate, and noninvasive clinical tool for assessment of FAI, and further, a means for planning of surgical interventions [10]. Advances in imaging techniques nowadays enable use of low-dose CT, with radiation levels comparable to radiography [11]. In 2015, a cadaveric study was performed to evaluate the ability of a simulation software to determine deformities limiting hip joint dynamics based on CT imaging [12]. The technique demonstrated potential to serve as a clinical diagnostic tool for patients presenting with FAI symptoms, as it provides a hypothetical ROM based on morphology. However, among patients with FAIS it remains unclear to which extent ROM is restricted due to morphology or by other pain sources. This feasibility study aimed to explore associations between ROM measured using CT motion simulation and maximum passive ROM measured clinically using 3D motion analysis in patients with FAIS, prior to and post arthroscopic hip surgery.

## Materials and methods

The study was reported following the “Strengthening the Reporting of Observational Studies in Epidemiology” (STROBE) statement as a guideline [13]. The study was approved by the regional ethical review board in Stockholm, Sweden, (DNR 015/467–31) and Radiation Protection Committee (Strålskyddskommittén) at Karolinska University Hospital (DNR K1031-2015). Eight male study participants subject to hip arthroscopy were prospectively recruited from Capio Artro Clinic, Sophiahemmet, Stockholm, Sweden between September 2015 and August 2016. Inclusion criteria included a typical history of FAIS, radiographic findings of cam FAI with an alpha angle > 60 degrees, with or without signs of pincer, 15–40 years of age, symptom duration > 1 year, and being scheduled for arthroscopic hip surgery for FAIS (Table 1). Exclusion criteria included previous surgery of the hip, osteoarthritis (cartilage height < 2 mm on radiographic examination of the hip according to Tönnis et al. [14]), hip dysplasia (defined as Wiberg angle < 25 degrees), rheumatoid arthritis, neurological disease, Mb Perthes, and/or body mass index (BMI) > 30. All arthroscopic hip surgeries were performed at Capio Artro Clinic, Sophiahemmet, Stockholm, Sweden, by two senior consultant orthopedic surgeons.

**Table 1:** Demographics and characteristics of included study participants with femoroacetabular impingement syndrome

Participant ID	Sports activity	Preoperative radiographic findings							
		Alpha angle	Extrusion (%)	Tönnis angle	Sharp angle	Os acetabuli	Cross over sign	Lateral center edge (Wiberg angle)	Tönnis score
1 Left hip	Ice hockey	76	26	9	48	No	No	27	0
1 Right hip		78	28	9	48	No	No	25	0
2	Thai boxing	78	18	5	40	No	No	33	0
3 Left hip	Motocross	74	15	− 3	33	No	Yes	48	0
3 Right hip		74	13	− 3	35	No	Yes	48	0
4 Left hip	Kickboxing	70	17	0	40	No	No	34	0
4 Right hip		74	17	0	40	No	No	36	0
5	Basketball	74	17	2	43	No	No	36	0
6 Left hip	Alpine skiing	72	12	3	38	No	No	38	0
6 Right hip		68	15	3	38	No	No	38	0
7	Ice hockey	79	26	9	39	No	No	25	0
8	Running	76	15	1	41	No	Yes	46	0

**Fig. 1:**
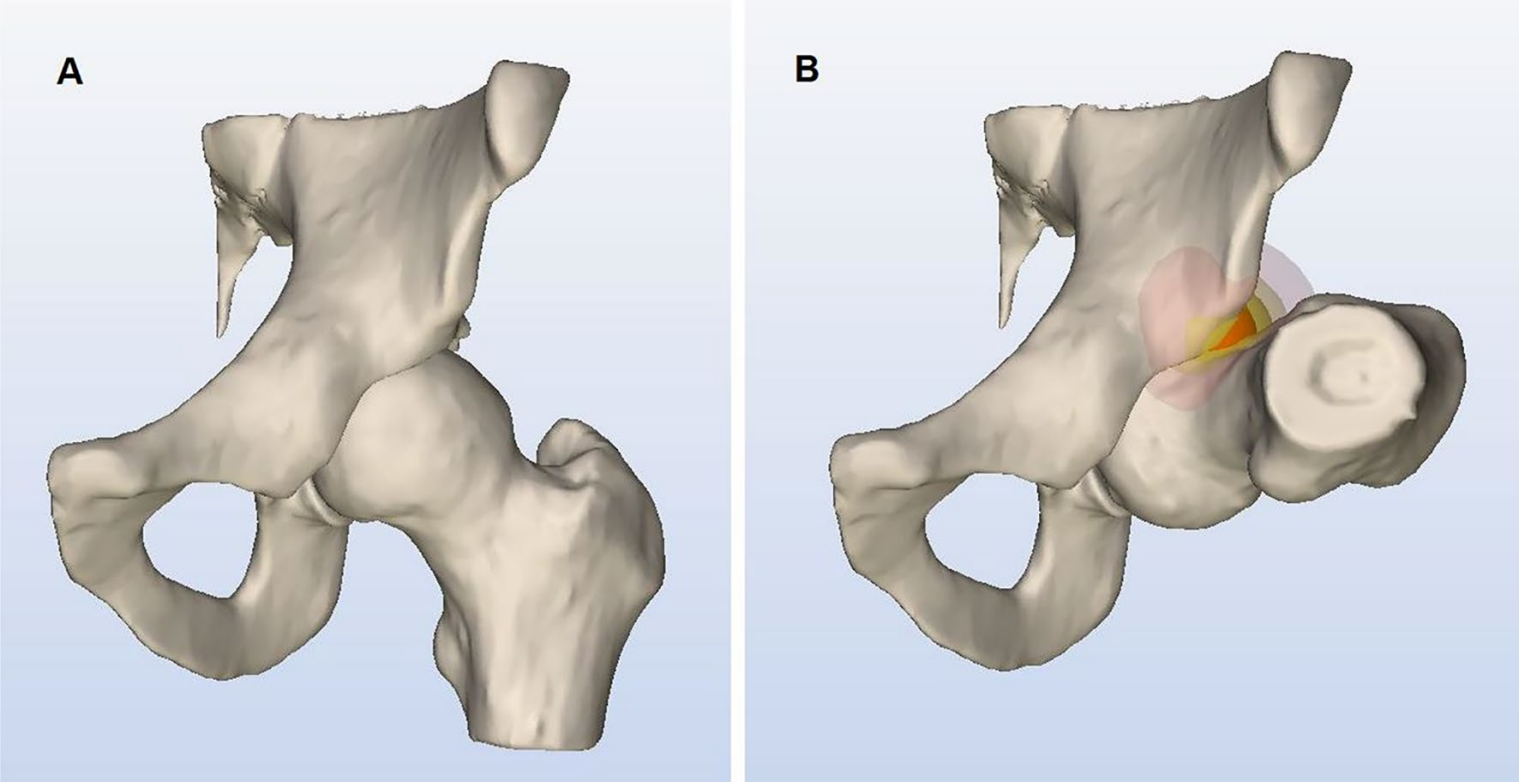
Computed tomography (CT) motion simulation using Articulis software (Clinical Graphics, Den Haag, The Netherlands). The software automatically converts CT scans to 3D models of the femur and pelvis. In this figure the hip joint is presented in an anterior–posterior projection with **a** the hip joint in neutral **b** the hip joint in 90 degrees’ flexion and 26 degrees’ internal rotation when the impingement occurs. The software identifies the impinging area by 0.1 mm

Hip joints of study participants were examined prior to and at median 7 months (range 4–11) following arthroscopic hip surgery using a Toshiba Dual-Energy CT at Karolinska University Hospital, Solna, Sweden, according to a low-dose protocol. Motion simulation was conducted using the simulation software, Articulis software (Clinical Graphics, Den Haag, The Netherlands). The software automatically converts the CT scans to 3D models of the femur and pelvis. The software identifies the impinging area by 0.1 mm and calculates the amount of bone necessary to resect to create a hip configuration without impingement (Fig. 1) [12]. Simulated values (measured in degrees) of maximum internal rotation with the hip in 90 degrees’ flexion, and maximum internal rotation plus adduction with the hip in 90 degrees’, respectively, were used in the analysis.

3D motion analysis was performed at the Motion Analysis laboratory at Karolinska University Hospital, Solna, Sweden, prior to and at median 7 months after surgery. Biomechanical measurements were obtained using an 8-camera system (Vicon Motion Systems Ltd, Oxford, UK). A conventional biomechanical model, the Plug-in-Gait model, including 18 reflective markers was used. Good intra-sessional repeatability has been reported using the Plug-in-Gait model [15], and for global kinematic data in healthy adults an inter-sessional standard error of 1.8 degrees [16]. Calculations of 3D motion analysis data were performed using MATLAB® software R2014a (The MathWorks, Inc., Natick, MA). Passive ROM of the hip joint was examined with the participant in a supine position, with reflective markers on, to record hip kinematics (i.e. maximum ROM of body segments) in three planes; frontal, sagittal and transversal. One examiner fixated the participant’s hip manually in 90 degrees’ flexion. A second examiner passively moved the joint towards maximum internal rotation. Next, with the hip still manually fixated in 90 degrees of flexion, maximum internal rotation plus adduction was evaluated. Study participants were asked to rate their perceived pain during the test of passive ROM using a visual analog scale (VAS). The VAS is a commonly used psychometric response scale for assessing pain, consisting of a 10 cm long line where the participant indicates how much pain he or she is experiencing [17]. It is measured in millimeters were 0 means “No pain at all” and 100 mm mean “Worst imaginable pain”.

### Statistical analysis

**Fig. 2:**
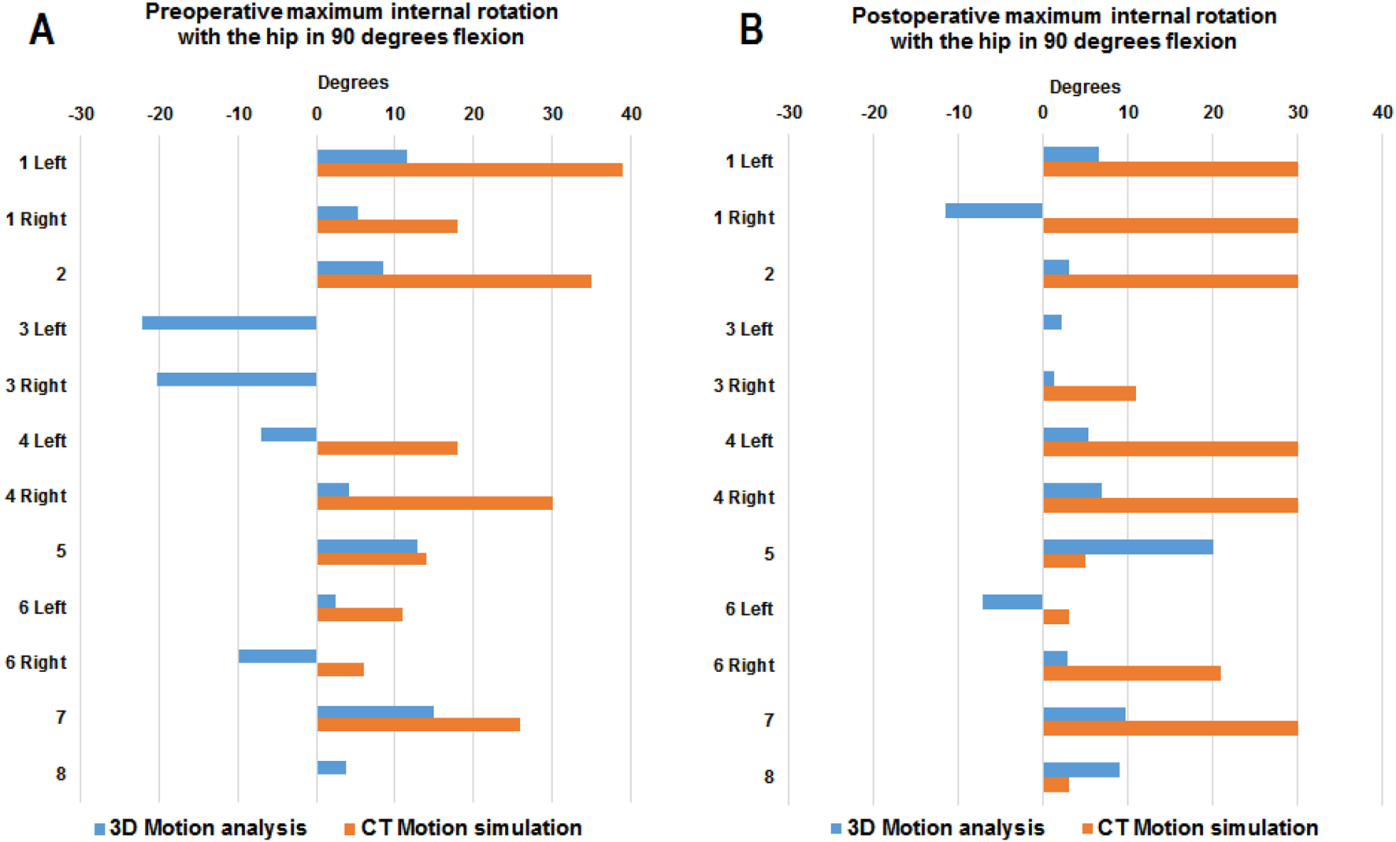
Maximum passive range of internal rotation with the hip in 90 degrees’ flexion measured using computed tomography (CT) motion simulation and three-dimensional (3D) motion analysis **a** prior to and **b** at mean 7 months after arthroscopic surgery. For participants with no orange bar, the CT simulation yielded 0 degrees of internal rotation with the hip in 90 degrees’ flexion

**Fig. 3:**
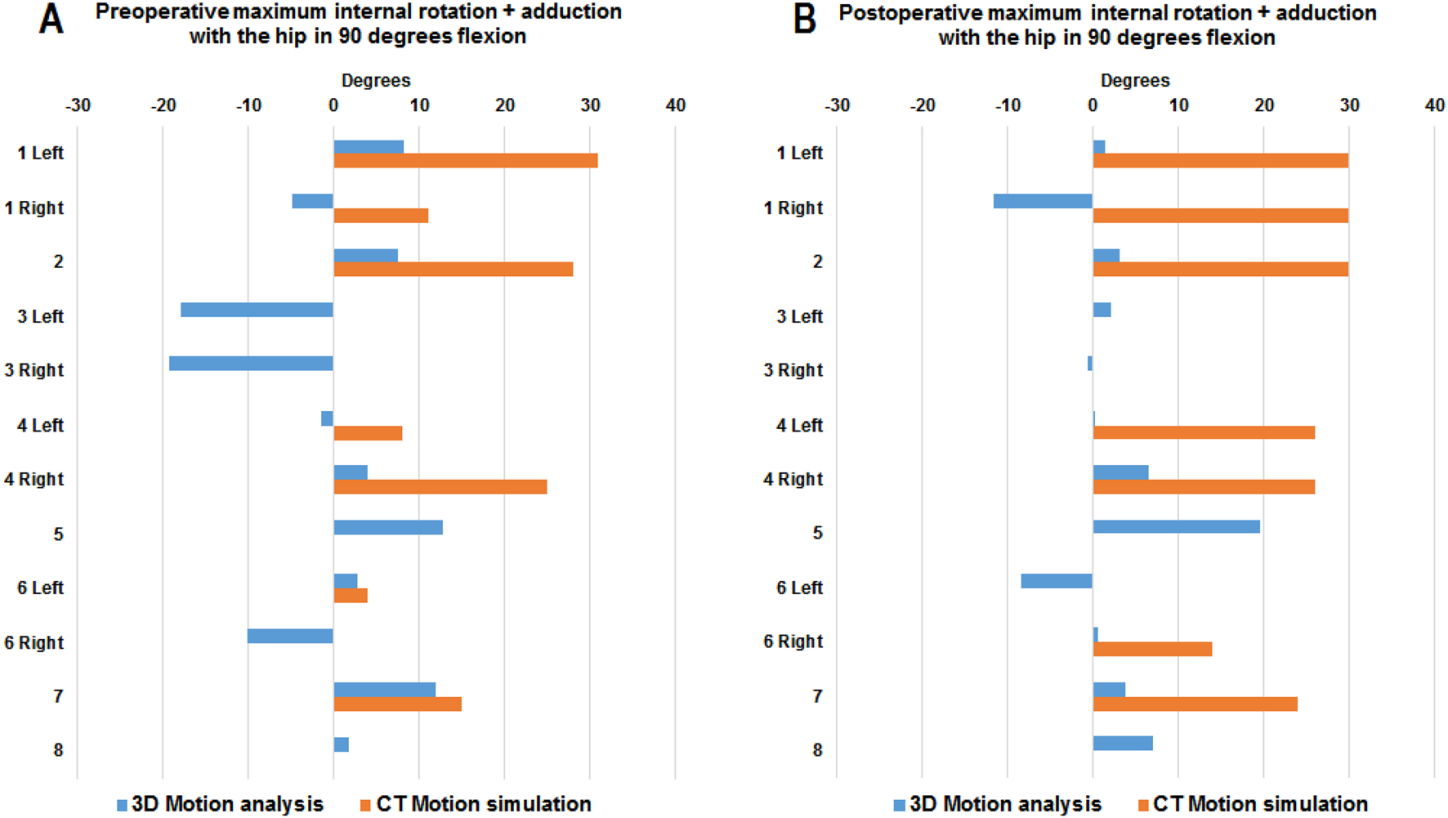
Maximum passive range of internal rotation *and* adduction with the hip in 90 degrees’ flexion measured using computed tomography (CT) motion simulation and three-dimensional (3D) motion analysis **a** prior to and **b** at mean 7 months after arthroscopic surgery. For participants with no orange bar, the CT simulation yielded 0 degrees of internal rotation with the hip in 90 degrees’ flexion and adduction

Due to the nature of this explorative feasibility study, no sample size calculation was performed prior to the study. Statistical analyses were performed using IBM SPSS Statistics version 26 (Chicago, IL). A significance level was set at ɑ = 0.05. Mean, standard deviation, median, range, and percentage described variables. Spearman rank correlation coefficients examined the strength of the relationship between ROM measured using CT motion simulation and 3D motion analysis [18]. Correlation coefficients were interpreted according to Dancey and Reidy, where an r-value of ± 1 is considered a perfect correlation, ± 0.7 to ± 0.9 strong, ± 0.4 to ± 0.6 moderate, ± 0.1 to ± 0.3 weak, and 0 inferring no correlation [19]. In case of a significant correlation, the relationship between methods was explored using univariate linear regressions to provide an estimate of the amount the dependent variable (3D motion analysis) increase with one unit increase in the independent variable (CT motion simulation) [20]. Paired sample t-tests were used to evaluate pre- to postoperative changes in ROM on group level. Differences in absolute ROM, prior to and after surgery, are presented and illustrated graphically (Figs. 2 and 3). Change in VAS pain, prior to and after surgery, was evaluated using Wilcoxon Signed Rank test.

## Results

### Demographics of included study participants

Eight males with a mean age of 27.2 years (SD 7.3), mean height 176 cm (SD 7.7), and mean BMI 21.1 (SD 2.1), were included in the study. Four of the included participants had bilateral FAIS and thus, in total, measurements from 12 hip joints were examined and included in the analysis (Table 1). Surgical resection of cam impingement was performed in all 12 examined hip joints, and surgical resection of pincer impingement was performed in three hip joints (Table 2). No labrum sutures were performed.

**Table 2:** Peroperative findings and surgical procedures performed

Participant ID	Cartilage damage (According to Konan 1A–4B)	Surgical procedures performed
1 Left hip	Konan 4B	Cam resection, micro fracturing of cartilage damage
1 Right hip	Konan 2A	Cam resection
2	Konan 4B	Cam resection, cartilage debridement
3 Left hip	Konan 1B	Cam resection, pincer resection
3 Right hip	Konan 4A	Cam resection, pincer resection
4 Left hip	Konan 2B	Cam resection
4 Right hip	Konan 2B	Cam resection
5	Konan 3A	Cam resection
6 Left hip	Konan 1A	Cam resection
6 Right hip	Konan 1A	Cam resection
7	Konan 3A	Cam resection
8	Konan 1A	Cam resection, pincer resection

Cartilage damage according to Konan [38]: grade 1, softening or wave sign; grade 2, cleavage lesion; grade 3, delamination; and grade 4, exposed bone. The site of the lesion is further classed as A, B or C

### Correlation between preoperative range of motion

Prior to surgery, the correlation between maximum internal rotation measured using CT motion simulation and 3D motion analysis was strong (Table 3). Linear regressions demonstrated that maximum internal rotation measured using CT motion simulation was predominantly larger than when measured using 3D motion analysis (Table 4, Figs. 2 and 3). On average, the difference between CT motion simulation and 3D motion analysis was 16 degrees (Table 4). No correlation between methods was found when preoperative maximum internal rotation coupled with adduction was examined (Table 3).

**Table 3:** Spearman rank correlations between maximum range of motion measured using computed tomography (CT) motion simulation and three-dimensional (3D) motion analysis in patients with femoroacetabular impingement syndrome, prior to and after arthroscopic hip surgery

Number of observations for each method (*n* = 12)	3D motion analysis	
CT motion simulation	*r*	*p*-value
*Test occasion and direction of passive range of motion*		
Preoperative hip flexion and internal rotation	0.71	< 0.01
Preoperative hip flexion, internal rotation and adduction	0.52	0.08
Postoperative hip flexion and internal rotation	0.14	0.66
Postoperative hip flexion, internal rotation and adduction	− 0.18	0.56

**Table 4:** Univariate linear regression examining agreement between maximum internal hip rotation measured using computed tomography motion simulation (independent variable) and three-dimensional motion analysis (dependent variable)

Model	Adjusted *r*^2^	Model *p*-value	Unstandardized coefficient Beta	Variable *p*-value	95% CI
Range of internal hip rotation	0.43	0.012	16.2	< 0.001	[9.5, 22.9]

### Correlation between postoperative range of motion

At a median duration of 7 months after arthroscopic hip surgery, no correlations were found between ROM measured using CT motion simulation and 3D motion analysis (Table 3).

### Pre- to postoperative change in range of motion

After surgery, the majority of included participants displayed increased ROM of the hip based on CT motion simulation and passive ROM of the hip measured using 3D motion analysis (Figs. 2 and 3). On group level, irrespective of evaluation method and movements examined, no significant pre-to postoperative changes in hip joint ROM were found after surgery (Table 5).

**Table 5:** Evaluation of pre-to-postoperative change in range of internal hip rotation

Number of observations for each method (*n* = 12)	Degrees of internal hip rotation			*p*-value	95% CI
	Preoperative evaluation	Postoperative evaluation	Pre-to postoperative change in range of internal hip rotation		
CT motion simulation	Mean (SD)				
The hip in 90 degrees’ flexion	16.4 (13.7)	18.6 (13.0)	2.2 (8.8)	0.412	[− 7.8, 3.4]
The hip in 90 degrees’ flexion + adduction	10.2 (11.9)	15.0 (13.9)	4.8 (8.0)	0.060	[− 9.9, 0.2]
3D motion analysis					
The hip in 90 degrees’ flexion	0.3 (8.0)	4.0 (8.0)	3.7 (12.6)	0.330	[− 11.7, 4.3]
The hip in 90 degrees’ flexion + adduction	− 0.4 (10.8)	1.9 (7.8)	2.3 (10.4)	0.452	[− 8.9, 4.3]

### Perceived pain

At the preoperative test session of clinically assessed ROM, study participants rated their perceived pain on the VAS to median 65 mm (range 20–93). After surgery, pain was significantly reduced to median VAS 28 mm (range 0–65) (*p* = 0.005).

## Discussion

This study aimed at exploring associations between bone-on-bone morphology-based hip joint ROM and pain restricted ROM in individuals with FAIS prior to and following arthroscopic hip surgery. To this end, range of internal hip rotation, and internal hip rotation coupled with adduction, was assessed using CT motion simulation and clinically using 3D motion analysis. The results demonstrated a strong correlation between the methods prior to surgery when maximum internal rotation was assessed. Linear regressions further demonstrated that internal hip rotation measured using CT motion simulation was predominantly larger than clinically assessed pain restricted ROM. When maximum internal hip rotation was combined with hip adduction no correlations were found. After surgery, no linear relationship between methods was found, this with respect to both maximum internal rotation, as well as internal rotation coupled with adduction.

Partly corroborating findings of the present study, Bedi et al., compared clinician measured ROM and CT based computer model estimated ROM in 10 hip joints with FAI, pre and post arthroscopic osteoplasty [21]. Results demonstrated significant correlations between the clinical measurements and the ROM predicted by the model. In contrast to results of the present study, Bedi and colleagues demonstrated that both hip flexion and internal rotation ROM were improved postoperatively, according to both the clinical assessment and the computer model. The authors conclude that, in symptomatic patients, the magnitude of improvement in ROM is not predictable based on radiographic measures alone. However, CT based computer modeling can be utilized to localize regions of anticipated mechanical impingement [21].

Prior to surgery, CT simulated internal hip rotation was predominantly larger than clinically assessed passive internal hip rotation measured using 3D motion analysis. On average, CT motion simulation yielded a ROM 16 degrees larger than 3D motion analysis. The simulated range of motion based on CT scans describe a theoretically possible ROM, allowed by the patient’s bone conformation, while for clinically assessed passive ROM, movement is affected by pain, discomfort, and possibly the labrum [22]. CT motion simulation may oversimplify hip contact mechanics, since these models include bone-to-bone contact but remove all soft tissue, including the cartilage and labrum [22]. Findings of a recent study, examining the effect of inclusion of the acetabular labrum on maximum ROM during simulation of the flexion–adduction–internal rotation impingement, demonstrated reduced internal rotation ROM by close to 20° and increased variability in the location of contact relative to the acetabular rim [22]. Many patients with FAIS have concomitant hip and groin pain originating from other sources, i.e. adductor-related pathology and soft tissues [23, 24] (i.e. muscles, tendons, ligament, and joint capsule), which may be contributing limiting factors. In addition, cartilage injuries and degenerative changes on joint surfaces commonly seen with FAIS also contribute to pain and limitations in function [25]. Consequently, deciphering the root of pain is difficult in FAIS.

No correlation between CT motion simulation and 3D motion analysis was found when internal rotation plus adduction was evaluated, although measurements were more aligned between methods at the preoperative assessment than at the postoperative assessment (preoperative: r = 0.52, *p* = 0.08, postoperative: r =  − 18, *p* = 0.56). Evaluation of hip joint range of motion in all three planes simultaneously is complex and difficult to measure clinically. In combination with additional stress on soft tissues, this could be a contributing factor to the statistically non-significant correlations found when adduction was added as compared to just flexion and internal rotation. This may further be reflected in the larger discrepancy seen postoperatively between the simulated and clinically assessed ROM (Fig. 3) where response to surgery is yet an influential factor on clinically assessed ROM. The comprehensive movement performed; hip flexion, adduction, and internal rotation (FADIR) is a commonly used examination test for FAI [26]. The test is considered positive if pain in end range movement occurs. However, the FADIR test has been reported to render a large number of false positive tests among adolescent ice hockey players without diagnosed hip disorder, and thus be inadequate for screening cam and pincer morphology [27]. Palmer et al. evaluated outcomes 8 months after arthroscopic hip surgery for FAIS and compared to physiotherapy and activity modification in a pragmatic randomized controlled study [28]. The authors found that patients treated with arthroscopic surgery displayed increased hip flexion postoperatively, however, remained fairly unchanged in hip adduction and internal rotation. At the 8-month follow-up after surgery, 63% of patients had a positive FADIR test [28]. In the light of previous research, where a false positive FADIR test is common in athletes without hip disorder, as well as a common finding in individuals with FAIS after surgery, the absent correlation between CT motion simulation and clinically assessed FADIR should not come as a surprise, neither preoperatively nor postoperatively.

After surgery, the majority of included patients displayed increased ROM of the hip based on CT motion simulation and passive ROM of the hip measured using 3D motion analysis. However, a few individuals demonstrated unchanged ROM after surgery, both according to CT motion simulation, and 3D motion analysis. Irrespective of evaluation method used, or movement combinations evaluated, no significant increase in range of motion on group level was observed postoperatively. After surgery, the number of individuals with a simulated (hypothetical) full range of internal rotation (i.e. 30 degrees) was greater when measured with hip flexion only, and not coupled with adduction. This result may reflect that it is not always technically possible to resect the entire bony prominence of the femoral head circumference to create a hip configuration without impingement using arthroscopy.

In the present study, ROM was re-evaluated at median 7 months after surgery. The timing of the postoperative evaluation may have impacted the results as pain was reduced, yet still present during test of passive maximal ROM. Preoperatively, study participants rated their perceived pain to be median VAS 65 (range 20–93). After surgery, pain was significantly reduced, although still present (median VAS 28, range 0–65). An alternative approach, which possibly could have rendered a larger ROM during the clinical assessment, and perhaps a stronger correlation between methods, would have been to inject the hip joint with local anesthesia. In line with our results, a recent cross sectional study evaluating objective hip-related function in patients following hip arthroscopy reported less hip mobility 6 to 10 months after surgery as compared to controls [29]. Previous studies evaluating patient-reported outcomes, including pain measurements, following hip arthroscopy report improvements at one and two years after surgery [30–34], indicating postoperative improvements beyond the time span evaluated in the present study. Furthermore, a recent meta-analysis evaluating return to sports following hip arthroscopy (for FAIS) concluded that the observed mean duration for return to play was 7.4 months [35]. Consequently, timing of the follow-up in the present study may be considered too early after surgery in a pain-perspective, yet reasonable in a functional mobility perspective.

This study holds several limitations. The study is based on a small number of observations, consequently, the risk for type two errors is evident, and the external validity as well as generalizability of the results are limited. The time period to follow-up may be considered too short as previously discussed, as further improvements during the first year postoperatively may be expected [30, 31]. The present study used 3D motion analysis to quantify the clinically assessed ROM, a method associated with limitations of its own. The biomechanical model Plug-in-Gait was used, which relies on anthropometric based regression equations to estimate the hip joint center [36]. The calculation is based on the width of the pelvis (to define the medial–lateral position of the hip joint center), and the distance between the anterior superior iliac and the greater trochanter [36]. Difficulties may occur when reflective markers cannot be placed directly on to the anterior superior iliac due to soft tissue. An alternative approach would have been to use a functional calibration method, where the hip joint center can be estimated from the movement of the thigh with respect to the pelvis during calibration trials [37]. However, in the present study, all included study participants had a BMI below 25 and no excessive soft tissue around the pelvis, hip or thigh. The movements examined were carried out passively by an examiner at slow speed, consequently, movement artefacts of the reflective markers due to soft tissue was not a problem. It was one and the same investigator placing the reflective markers for all 3D motion analyses, and the errors related to the biomechanical model did not change between pre- and post-evaluation, and should therefore, not have a huge impact on the results. Nevertheless, the estimated hip joint center may affect the measured ROM.

## Conclusion

Findings of this study suggest that ROM is restricted by pain to a greater extent than mechanical, bone-on-bone, morphology-based impingement in individuals with FAIS both pre- and postoperatively. Prior to arthroscopic hip surgery, the correlation between clinically assessed maximum internal hip rotation and ROM measured using CT motion simulation is strong. CT motion simulation, exclusive labrum, cartilage and soft tissues, indicates a greater theoretically possible ROM, however, postoperatively no statistically significant correlation to clinically assessed ROM was found, indicating less predictable postoperative results on pain and function.

## Data Availability

The datasets that are used and analyzed for the present study are available from the corresponding author on reasonable request.
